# Extent of polymorphism and selection pressure on the *Trypanosoma cruzi* vaccine candidate antigen Tc24

**DOI:** 10.1111/eva.13068

**Published:** 2020-09-10

**Authors:** Audrey Arnal, Liliana Villanueva‐Lizama, Christian Teh‐Poot, Claudia Herrera, Eric Dumonteil

**Affiliations:** ^1^ Laboratorio de Parasitología Centro de Investigaciones Regionales “Dr Hideyo Noguchi” Universidad Autónoma de Yucatán Mérida Mexico; ^2^ Departamento de Ecología de la Biodiversidad Instituto de Ecología Universidad Nacional Autónoma de México Ciudad de México México; ^3^ Department of Tropical Medicine School of Public Health and Tropical Medicine Tulane University New Orleans LA USA; ^4^ Vector‐Borne and Infectious Disease Research Center Tulane University New Orleans LA USA

**Keywords:** antigenic variation, calcium‐binding, Chagas disease, kinetoplastid, vaccine

## Abstract

**Introduction:**

Chagas disease, caused by the protozoan parasite *Trypanosoma cruzi*, is a major public health problem in the Americas, and existing drugs have severe limitations. In this context, a vaccine would be an attractive alternative for disease control. One of the difficulties in developing an effective vaccine lies in the high genetic diversity of *T. cruzi*. In this study, we evaluated the level of sequence diversity of the leading vaccine candidate Tc24 in multiple parasite strains.

**Methods and Results:**

We quantified its level of polymorphism within and between *T. cruzi* discrete typing units (DTUs) and how this potential polymorphism is structured by different selective pressures. We observed a low level of polymorphism of Tc24 protein, weakly associated with parasite DTUs, but not with the geographic origin of the strains. In particular, Tc24 was under strong purifying selection pressure and predicted CD8^+^ T‐cell epitopes were mostly conserved. Tc24 strong conservation may be associated with structural/functional constrains to preserve EF hand domains and their calcium‐binding loops, and Tc24 is likely important for the parasite fitness.

**Discussion:**

Together, these results show that a vaccine based on Tc24 is likely to be effective against a wide diversity of parasite strains across the American continent, and further development of this vaccine candidate should be a high priority.

## 1 INTRODUCTION

Chagas disease is a chronic disease caused by the protozoan parasite *Trypanosoma cruzi*, which is mainly transmitted to humans and other mammals through contaminated feces of hematophagous bugs called triatomines (family Reduviidae). Less commonly, *T. cruzi* is transmitted congenitally, through blood transfusion and transplantation, or from consumption of contaminated food or beverages (Rassi, Rassi, & Marin‐Neto, 2010; WHO, 2017). Chagas disease is a major public health problem in Latin America where it is estimated that at least six to seven million people are currently infected, causing incapacity in infected individuals and more than 10,000 deaths per year (WHO, 2017).

The initial acute phase of Chagas disease develops 1–2 weeks after the infection and can be characterized by an elevated parasitemia associated with nonspecific signs and symptoms such as fever (Benck, Kransdorf, & Patel, 2018). Approximately 20%–30% of individuals infected with *T. cruzi* progress to the chronic stage and develop cardiomyopathy or more rarely megacolon or megaesophagus (Benziger, do Carmo, & Ribeiro, 2017; Rassi et al., 2010; Ribeiro, Nunes, Teixeira, & Rocha, 2012). The current etiological treatments for Chagas disease, benznidazole or nifurtimox, result in a reduction in detectable parasitemia (Bern, 2011). However, these drugs can have severe side effects and limited efficacy has been demonstrated in adults and advanced chronic patients (Morillo et al., 2015; Pecoul et al., 2016; Pérez‐Molina et al., 2009). In this context, a vaccine would be an attractive and cost‐effective alternative to improve the control of Chagas disease (Beaumier, Gillespie, Hotez, & Bottazzi, 2013; Beaumier et al., 2016; Dumonteil et al., 2012).

Vaccination has the advantages of relying on short administration regimens, and the induction of multiple effector mechanisms against the pathogen may have high efficacy to control the infection and lower the possibilities of resistance (Bahloul et al., 2003; Boyer et al., 1997; Lai, Pakes, Ren, Lu, & Bennett, 1997; Lodmell & Ewalt, 2001; Lowrie et al., 1999). During the last decade, several vaccine types have been found immunogenic and protective in mouse models, providing proof‐of‐concept data on the feasibility of a preventive or therapeutic vaccine to control a *T. cruzi* infection (see for review Quijano‐Hernandez & Dumonteil, 2011). However, one of the difficulties in developing an effective vaccine lies in the high levels of genetic variability of *T. cruzi*, which may lead to antigenic variability and immune evasion of some parasite strains (Haolla et al., 2009). Indeed, *T. cruzi* has been divided into seven discrete typing units (DTUs, TcI‐VI) based on molecular markers (Telleria & Tibayrenc, 2017; Zingales et al., 2012), including two hybrid lineages (TcV and TcVI), and one found mostly in bats (TcBat) (Marcili et al., 2009; Ramírez et al., 2014). Therefore, it remains essential to identify how this genetic diversity is distributed in the endemic regions and to consider its impact on antigenic diversity for vaccine and diagnostic development.

The antigen Tc24 is one of the leading candidates for an immunotherapeutic vaccine against *T. cruzi* (Dumonteil et al., 2012; Gunter et al., 2016; Sanchez‐Burgos et al., 2007). This protein of 24 kDa is ubiquitously expressed in all stages of *T. cruzi* strains (Guevara, Taibi, Billaut‐Mulot, & Ouaissi, 1997; Umezawa et al., 1999) from multiple gene copies located in tandem arrays (Porcel et al., 1996). It has calcium‐binding domains and is localized in the flagellar pocket (Hopkins et al., 2011; Ouaissi, Da Silva, Guevara, Borges, & Guilvard, 2001; Ouaissi et al., 1990). It is an immune modulator and possesses B‐cell superantigenic properties (Cordeiro Da Silva, Espinoza, Taibi, Ouaissi, & Minoprio, 1998). This antigen facilitates immune escape by interfering with antibody‐mediated responses, particularly the avoidance of catalytic antibodies (Gunter et al., 2016). These antibodies are an innate host defense mechanism present in the naive repertoire, and catalytic antibody–antigen binding results in hydrolysis of the target (Gunter et al., 2016). The therapeutic administration of a DNA vaccine encoding Tc24 can stimulate the immune response and lead to the control of disease progression in murine and canine models of *T. cruzi* infection (Limon‐Flores et al., 2010; Quijano‐Hernandez, Bolio‐González, Rodríguez‐Buenfil, Ramirez‐Sierra, & Dumonteil, 2008; Sanchez‐Burgos et al., 2007). We have thus developed a recombinant protein expression system for the production of this vaccine candidate (Barry et al., 2019; Villanueva‐Lizama et al., 2018), and recombinant Tc24 protein in multiple formulations can decrease parasitemia and cardiac parasite burden in immunized mice compared to controls (Dumonteil, Escobedo‐Ortegon, Reyes‐Rodriguez, Arjona‐Torres, & Ramirez‐Sierra, 2004; Martinez‐Campos et al., 2015; Sanchez‐Burgos et al., 2007). Further enhancements of this vaccine candidate include the mutagenesis of four cysteine residues, which facilitates the production process of Tc24‐C4 while maintaining its immunogenicity and protective efficacy (Biter et al., 2018).

Therefore, the Tc24 antigen appears as a promising vaccine candidate, but little is known about the extent of its genetic variability among parasite strains. An initial study indicated about 97% sequence conservation of the Tc24 amino acid sequences among five *T. cruzi* strains from TcI, TcII, and TcVI DTUs (Dm28c, SilvioX10, Y, Tulahuen and CL) (Maldonado et al., 1997). In addition, Tc24‐like genes are found in other species such as *Trypanosoma conorhini*, *Trypanosoma freitasi*, *Trypanosoma lewisi*, *Herpetomonas megaseliae*, *Leptomonas seymouri*, and *Phytomonas serpens* (Maldonado et al., 1997).

Accordingly, the aim of this study was to evaluate in detail the extent of Tc24 diversity in multiple *T. cruzi* parasite strains and DTUs. To do so, we quantified its level of polymorphism within and among *T. cruzi* DTUs from multiple countries, and evaluated how its polymorphism may be structured by selective evolutionary pressures. Such analyses have been found important to assess forces driving protein evolution (Bitencourt Chaves et al.., 2017; Kumar et al., 2018).

## 2 MATERIALS AND METHODS

### 2.1 Tc24 sequences

Raw sequence reads from whole genome sequencing projects from 32 *T. cruzi* strains were obtained from the NCBI Sequence Read Archive database for analysis, as well as five annotated genome sequences obtained from the TriTryp database (Table 1). These strains covered TcI to TcVI DTUs, although TcI was over‐represented, and originated from multiple countries across the Americas.

**TABLE 1:** List of *Trypanosoma cruzi* strains

Strains	DTU	Country of origin
Arequipa	TcI	Peru
Bug2148^a^	TcI	Brazil
CGl14	TcI	Colombia
Corpus Christi	TcI	USA
Dm28c^a^	TcI	Colombia
H1b	TcI	Mexico
H2	TcI	Panama
H3	TcI	Panama
H5	TcI	Panama
H6	TcI	Panama
H7	TcI	Panama
H9	TcI	Panama
H12	TcI	Panama
H14	TcI	Panama
H15	TcI	Panama
Jose	TcI	Brazil
TBM3324	TcI	Ecuador
TBM3479B1	TcI	Ecuador
TBM3519W1	TcI	Ecuador
TBM3406B1	TcI	Ecuador
TD23	TcI	USA
TD25	TcI	USA
V1	TcI	Panama
V2	TcI	Panama
V3	TcI	Panama
X10462	TcI	Venezuela
X12422	TcI	Venezuela
Esmeraldo	TcII	Brazil
Y	TcII	Brazil
231^a^	TcIII	Brazil
M6241	TcIII	Brazil
CanIII	TcIV	Brazil
9280 cl2	TcV	Bolivia
CLBrener^a^	TcVI	Brazil
H1a	TcVI	Panama
TCC^a^	TcVI	Argentina
Tula cl2	TcVI	Chile

^a^ Indicates assembled genomes obtained from the TriTryp database.

Tc24 nucleotide sequences were extracted from the reads of *T. cruzi* strain genomes by rapid sequence mapping at a medium–low sensitivity using the software Geneious 9.1. The aligned Tc24 sequence reads were annotated for variants using the SNP/Free Bayes function of Geneious (Garrison & Marth, 2012). Every significant change in the sequences was recorded to generate lists of Tc24 nucleotide sequence variants for each *T. cruzi* strain. For analysis of copy number, we used the annotated genomes of Dm28c (TcI) and TCC strains (TcVI), which have been obtained by long‐read sequencing on a PacBio single‐molecular real‐time platform, and represent some of the most complete genome assemblies currently available for *T. cruzi* (Berná et al., 2018). These genomes were searched for Tc24 sequence using BLAST, and only matches including the full‐length coding sequence of Tc24 were considered. Similar BLAST searches of other assembled *T. cruzi* genomes were also performed. We calculated nucleotide diversity (π) and haplotype diversity (Hd) for Tc24 sequences. All the Tc24 nucleotide sequences were translated to the corresponding protein sequences using the software Geneious 9.1. Amino acid sequences were aligned using MUSCLE (Edgar, 2004a, 2004b), and phylogenetic trees were created using the Maximum‐likelihood as implemented in PhyML. To determine whether there is a DTU or country effect in structuring Tc24 protein diversity, we compared phylogenetic distances (pairwise genetic distances) among nodes within and between different groups through a nonparametric Wilcoxon test using R software 3.6.1. We further tested for a spatial structure by evaluating isolation by distance through a Mantel test with 10,000 permutations.

### 2.2 Analysis of selection pressures

Analysis of selection pressures on Tc24 nucleotide sequences was performed in MEGA software (10.0.4 version). We performed a single‐likelihood ancestor counting (SLAC) analysis, which uses a combination of maximum‐likelihood (ML) and counting approaches to infer nonsynonymous (dN) and synonymous (dS) substitution rates on a per‐site basis for a given coding alignment and corresponding phylogeny. This method assumes that the selection pressure for each site is constant along the entire phylogeny (Kosakovsky Pond & Frost, 2005), and statistical significance is ascertained at each site using an extended binomial distribution (Kosakovsky Pond & Frost, 2005). We also performed a McDonald–Kreitman (MK) test to assess selection among *T. cruzi* Tc24 genes (Egea, Casillas, & Barbadilla, 2008). For estimates of divergence, we used a closely related *T. rangeli* Tc24 sequence (accession #KC544829).

### 2.3 Epitope identification

We identified the Tc24 protein epitopes able to bind to the HLA‐I alleles reported as more frequent in the Mexican mestizo population (HLA‐A*02, A*24, B*35, and B*39) using SYFPEITHI, BIMAS‐HLA, IEDB, RANKPEP, PROPRED‐I, ANNPRED, COMPRED, SVMHC, PREDEP, and NETMHC algorithms. Predictions for HLA‐A*02 were included in 10 algorithms, for HLA‐A*24 in 5 algorithms, for HLA‐B*35 in 10 algorithms, and for HLA‐B*39 in 6 algorithms. For each HLA allele, the 10 best peptides predicted by each analysis program were selected according to their prediction value. A consensus analysis was carried out with the peptides selected for each allele, and we selected the peptides that were predicted by ≥5 programs for the HLA‐A*2 allele, for ≥3 programs for the HLA‐A*24 allele, ≥5 for the allele HLA‐B*35, and ≥3 for allele HLA‐B*39 (Teh‐Poot et al., 2015). Finally, the number of peptides that can be recognized by each of the alleles evaluated and their location in the amino acid sequence of the corresponding protein was determined (Doytchinova, Guan, & Flower, 2006).

### 2.4 Mapping of amino acid variants on 3D protein structure

We used the previously determined 3D structure of *T. cruzi* Tc24 protein (Wingard et al., 2008) to map the position of sites under significant selection pressure, and assess potential structural and functional constrains on the protein. Molecular graphics and visualization of residues under selection pressure were performed with UCSF Chimera (Pettersen et al., 2004).

## 3 RESULTS

**FIGURE 1:**
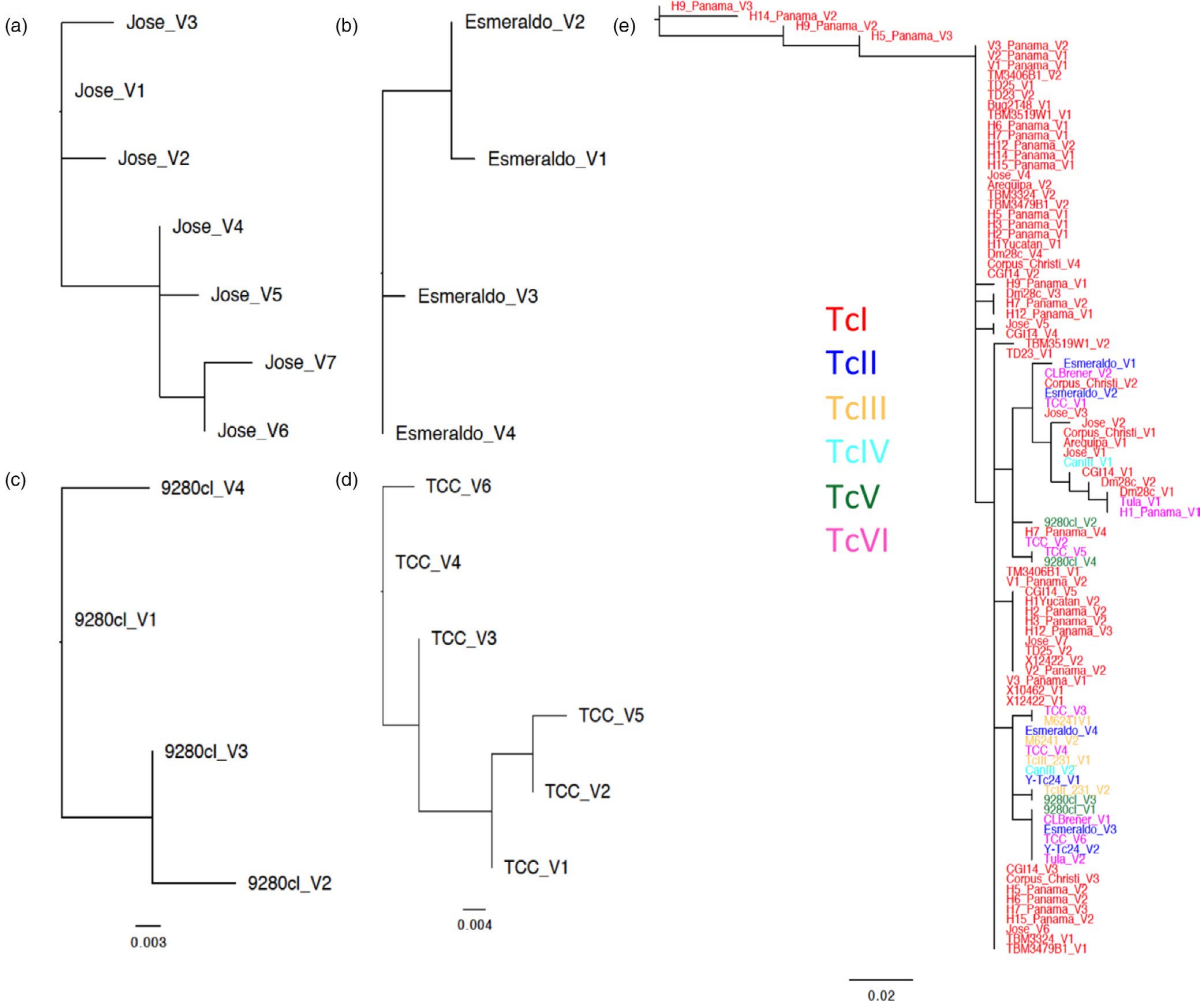
Relationships among Tc24 protein sequences. The phylogeny for Jose, Esmeraldo, 9,280 cl1, and TCC strains is shown in a–d, respectively. The phylogeny with all the strains (*N* = 37) is shown in (e). Each DTU is color‐coded as indicated. V1–V7 after strain names indicate sequence variants for each strain

We analyzed the full genome sequences currently available from 37 *T. cruzi* strains, to identify a total of 367 Tc24 nucleotide sequences, corresponding to 96 unique Tc24 protein sequences (211 amino acids) with 1 to 7 variant protein sequences per strain/genome. Most strains (28/37) had two sequence variants, two had a unique Tc24 protein sequence, and some (8/37) presented 3–7 sequence variants. Phylogenetic analysis of this intra‐strain sequence diversity showed two clear clusters of sequences, with a similar level of sequence diversity irrespective of the DTU of the strains (Figure 1a‐d). These data indicate a multicopy gene within a diploid genome, with limited sequence diversity among the respective gene copies within each genome. Indeed, further analysis of Dm28c (TcI) and TCC (TcVI) genome sequences indicated the presence of 60 copies of full‐length Tc24 genes in the haploid Dm28c genome and 43 copies in the diploid TCC genome, located in tandem arrays in two or three contigs, respectively.

Phylogenetic analysis of Tc24 sequence diversity among strains and DTUs indicated multiple clusters of sequences, indicating some level of sequence diversity (Figure 1e). Nucleotide diversity (π) was 0.00957 ± 0.00188, and haplotype diversity (Hd) was 0.958 ± 0.012. However, this genetic structuration was not associated with the geographic origin of the strains, and somewhat loosely with the DTUs, with four main clusters of sequences corresponding to TcI DTU, and two clusters of sequences from other DTUs. Statistical analysis of pairwise genetic distances among sequences further supported the lack of structure according to the country of origin (Wilcoxon test among countries, W = 4,948.5, *p* = .9). A Mantel test also indicated a lack of isolation by distance (*R* = −.0098, *p* = .48). On the other hand, there was a significant structuring according to DTUs (Wilcoxon test, W = 4,069.5, *p* = .022), although this was mostly due to differences between TcI and the other DTUs, as there was no structuring according to the DTU when excluding TcI (Wilcoxon test, W = 5,243, *p* = .55), which represented most (70/96) of the sequences.

**FIGURE 2:**
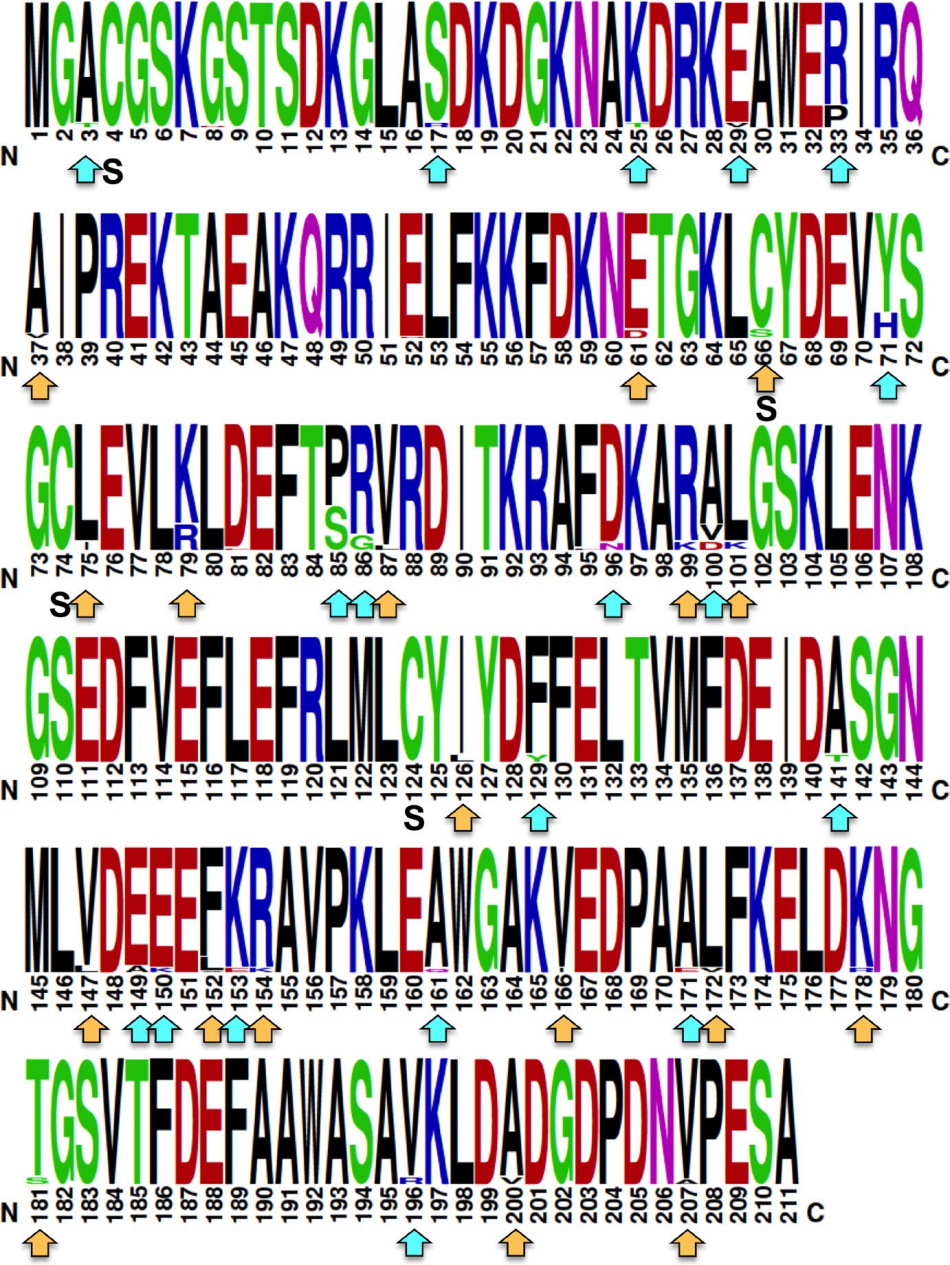
WebLogo of Tc24 protein sequence. Arrows under the sequence point to variant AA, with orange arrows for chemically similar AA and blue arrows for chemically different AA. S indicates C residues that have been mutated to S in the Tc24‐C4 vaccine candidate. Amino acids are colored according to their chemical properties: polar amino acids (G,S,T,Y,C,Q,N) are green, basic (K,R,H) are blue, acidic (D,E) are red, and hydrophobic (A,V,L,I,P,W,F,M) amino acids are black

We then analyzed in more detail how sequence variation was distributed within the Tc24 protein sequence. From the full protein sequence of 211 amino acids, sequence variation occurred at only 36 sites (17%), and the remaining amino acids were conserved (175/211, 83%) (Figure 2). Of the variant sites, only a few presented variant frequencies in over 2% of the sequences (sites 33, 71, 79, 85, and 86 for example). However, variant amino acids often had comparable physicochemical structures such as for site 66 or 79. Thus, Tc24 sequence diversity appeared to be focused on a limited number of sites within the protein, which is otherwise highly conserved among *T. cruzi* strains. The four cysteine residues that were mutated to serine in our vaccine antigen to facilitate its large‐scale production process (C4, C66, C74, and C124) corresponded to highly conserved residues.

**FIGURE 3:**
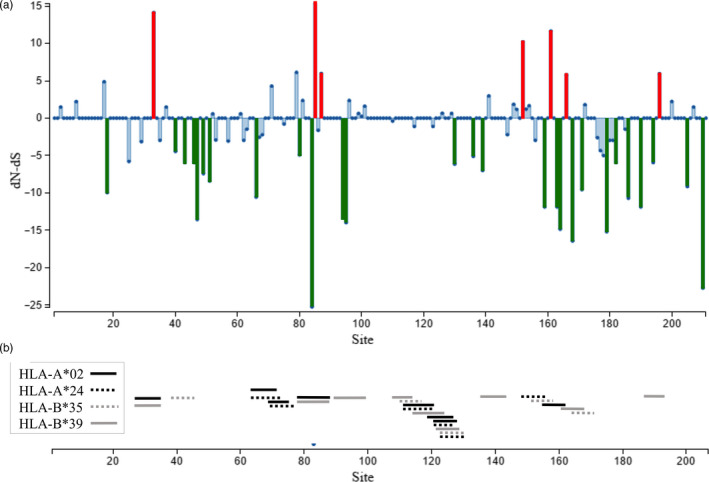
Selective pressure and CD8^+^ T‐cell epitopes in Tc24 antigen. (a) Selective pressures on Tc24 protein, expressed as dN‐dS ratio, were determined by SLAC analysis. Statistically significant selection pressure is highlighted in green (purifying selection) and red (diversifying selection), respectively. (b) Localization of the Tc24 protein epitopes with a high probability of binding to HLA‐I alleles. Horizontal lines correspond to epitopes for the indicated HLA alleles

We next analyzed potential selective pressures on Tc24 to understand what factors may drive its conservation among parasite strains and performed an analysis of synonymous and nonsynonymous mutations on our set of nucleotide sequences. Among the 211 codons of the Tc24 protein, 35 have a dN‐dS ratio significantly different from zero, indicative of selection pressure. The majority of these codons (28/35, 80%) presented an excess of synonymous substitutions and are under purifying (negative) selection, while only 7/35 (20%) showed excess nonsynonymous substitutions and are under diversifying (positive) selection (Figure 3a and Table S1). Thus, overall, the Tc24 protein appeared under strong purifying selective pressure, which may explain its limited level of polymorphism. We further performed a MK test of selection using a *T. rangeli* Tc24 sequence to estimate divergence and found a neutrality index of 0.788, with a proportion of adaptive substitutions (α) of 0.211 (χ^2^ = 0.33, *p* = .56). This indicated that Tc24 tended to present an excess of nonsilent divergence (as expected under positive selection) within *T. cruzi* species.

To further understand the selection pressure on Tc24, we assessed whether the codons under diversifying selection were located within potential epitopes with a high probability of HLA binding. We predicted 25 Tc24 protein epitopes with a high probability of binding to class I HLA alleles, with six binding to HLA‐A*2, six to HLA‐B*24, five to HLA‐B*35, and eight to HLA‐B*39 (Figure 3b). In addition, some epitopes were predicted to have a high probability of binding to more than one HLA allele, such as peptide RLDEFTSGV that can bind to alleles A*02 and B*39 and peptide EFLEFRLML that can bind to alleles A*02 and A*24. Furthermore, the protein sequence comprised between amino acids 109 and 136 included multiple overlapping predicted epitopes for several HLA alleles, which corresponds to a conserved region of the protein. Detailed analysis of 17 nonredundant predicted epitopes indicated that only four (23%) had amino acids subject to significant diversifying selection (Figure S1), while seven (41%) had amino acids subject to significant purifying selection, and an additional six (35%) were conserved but without significant selection. Thus, selection pressure for immune evasion could explain part of the diversifying selection detected on some of Tc24 residues. In addition, four predicted epitopes included a cysteine residue that was mutated to serine in Tc24‐C4 antigen, corresponding to C66 in one predicted epitope and C124 in three overlapping epitopes.

**FIGURE 4:**
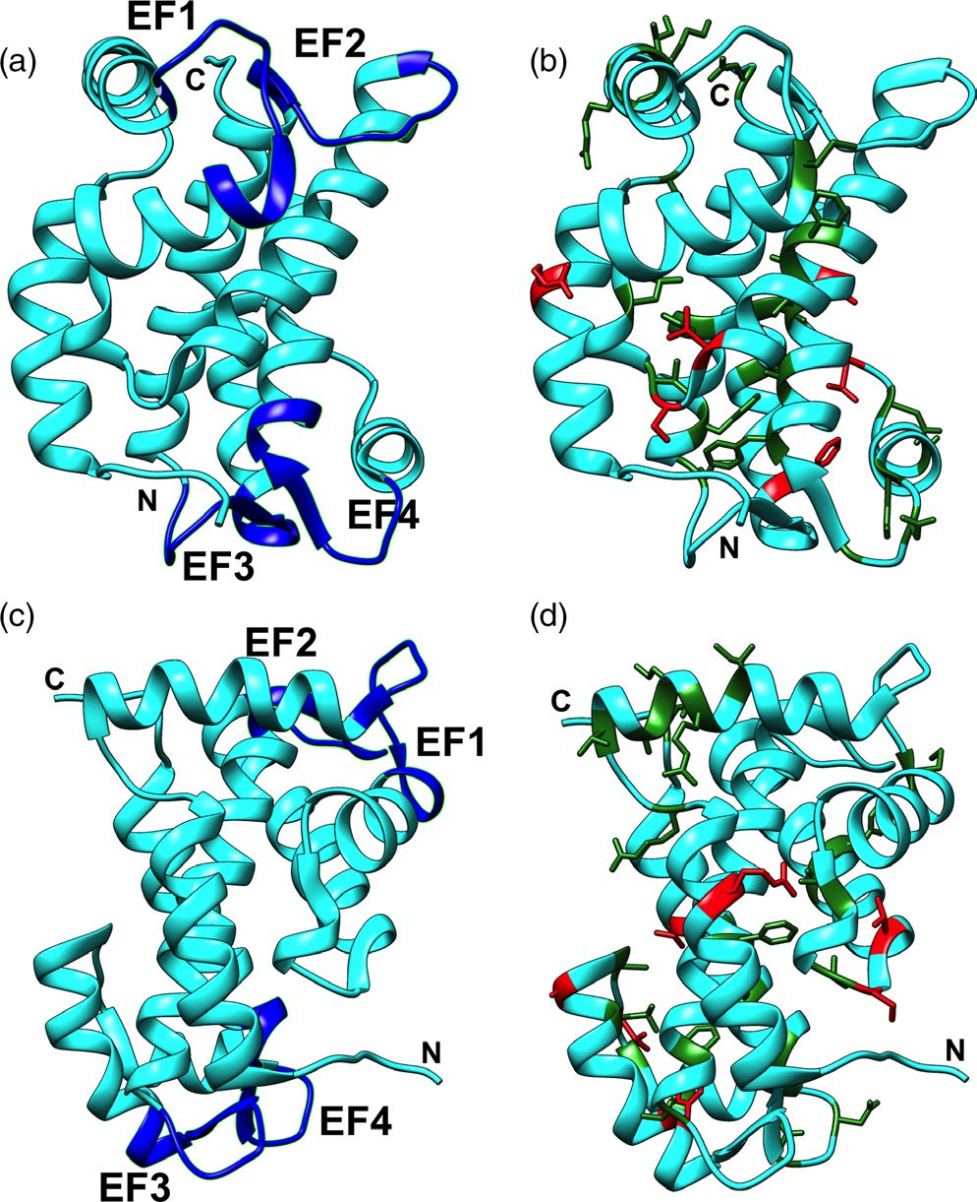
Location of selection pressures on the structure of the Tc24 antigen. EF hands are highlighted in blue (a and c). Sites under purifying selection are indicated in green, and sites under diversifying selection are indicated in red (b and d)

Finally, we assessed Tc24 structural/functional constrains that may contribute to the selection pressure detected on the protein by mapping the amino acids under purifying and diversifying selection onto the 3D structure of Tc24 (Wingard et al., 2008). Importantly, 17/35 sites under purifying selection (49%) were distributed within the four EF hand domains of the proteins, with four of these sites located within the Ca^2+^‐binding loops (Figure 4), suggesting some important constrains to conserve these functional domains. On the other hand, only one of the seven sites under diversifying selection (14%) was located in one of the EF hand domains (EF3), and the remaining six sites were spread within some of the α‐helices of Tc24, but not within these critical domains (Figure 4). Thus, functional/structural constrains on Tc24 protein appear to contribute at least in part to the overall strong purifying selection acting of the protein.

## 4 DISCUSSION

The development of an effective vaccine against *T. cruzi* needs to take into account the high levels of genetic variability of this parasite, as antigenic variability and immune evasion of some parasite strains may restrict the protective efficacy of a vaccine (Haolla et al., 2009). Nonetheless, limited studies have investigated the antigenic diversity of *T. cruzi* vaccine antigens (Knight, Zingales, Bottazzi, Hotez, & Zhan, 2014). Members of the trans‐sialidase family, the largest family of surface proteins of the parasite, were found to be under strong evolutionary pressure, likely from the immune system, for the selection of variants leading to immune evasion, and frequent recombination was identified as a contributing mechanism (Weatherly, Peng, & Tarleton, 2016). Diversifying selection and variant motifs within trypomastigote small surface antigen have also been identified, which has led to DTU‐specific serological diagnostic of the infection (Bhattacharyya et al., 2010). In this study, we evaluated the extent of polymorphism of one of the leading vaccine antigen, the flagellar‐associated calcium‐binding protein Tc24, among multiple *T. cruzi* strains from most of the American continent.

Our identification of Tc24 genes in the genomes of multiple strains of *T. cruzi* is in agreement with initial observations indicating that it is a multicopy gene located in tandem arrays (Porcel et al., 1996). We further identified 120 and 43 full‐length copies in Dm28c and TCC diploid genomes, respectively, indicating a significant variation in gene copy number among strains. Nonetheless, these genes encoded for a limited number of protein variants per strain, with only up to seven distinct Tc24 proteins encoded per genome. This limited diversity of Tc24 among gene copies within genomes suggests an important functional role of this protein in *T. cruzi* biology.

Phylogenetic analysis of Tc24 protein sequences from multiple parasite strains further indicated a low but significant sequence diversity among them. While no clear clustering of Tc24 sequences could be detected based on the geographic origin of the strains, some clustering seemed to exist among *T. cruzi* DTUs, with sequences from non‐TcI strains found in two clusters, while sequences from TcI strains were divided into four main clusters. This is in relative agreement with the currently accepted evolutionary history of *T. cruzi* DTUs, with TcI considered an ancestral DTU, and TcV and TcVI being hybrids derived from TcII and TcIII (Ramírez, Torres, Torres, & Curto, 2017). Further analysis with additional sequences from non‐TcI strains should help refine these phylogenetic relationships. Nonetheless, genetic diversity among Tc24 sequences was overall limited, with only 35/211 (17%) residues presenting some variants.

Further analysis indicated that Tc24 is under strong selective pressures, with the majority of the sites under significant purifying selection preventing any amino acid changes, and only a few sites subject to diversifying selection. Four predicted CD8^+^ epitopes had amino acids subject to significant diversifying selection, suggesting that Tc24‐specific immune responses may in part be driving this diversifying selection pressure, to allow parasites with sequence variants to escape the immune response. However, the majority of predicted epitopes were conserved, suggesting that the balance of diversifying and purifying selection pressures was biased toward purifying selection, favoring protein conservation. Topological evaluation of the distribution of selection pressure on the 3D structure of the protein suggested that constrains to preserve the structure and function of Tc24 four EF hand domains, which include calcium‐binding loops, may contribute to the strong purifying selection acting on the protein.

Taken together, these results emphasize that Tc24 is an excellent target antigen for vaccine development. Its low level of polymorphism, combined with the lack of structure according to geographic location, suggests that a vaccine based on this protein should be effective against a wide diversity of parasites circulating in the American continent. Moreover, the strong purifying selective pressures identified in this study demonstrate that this protein likely plays an important role in the parasite fitness, which strengthens the rationale for targeting the host immune response on this protein, as immune evasion would be less likely. Initial studies suggested that Tc24 may be used as antigen for the serological diagnostic of *T. cruzi* infection (Dumonteil et al., 2004; Villanueva‐Lizama et al., 2018), and it has been incorporated as part of recombinant antigen mixtures in some commercial tests.

Implementing a vaccine targeting this protein may impact the evolution of the parasite in the field and induce a vaccine escape phenomenon, with could produce potentially detrimental outcomes such as an increase in parasite virulence. Such a response has been already identified for various diseases (Kennedy & Read, 2017); it is therefore important to consider. One possibility could be to develop a “cocktail” vaccine targeting multiple proteins in order to distribute these selective pressures over multiple parasite antigens and therefore further reduce the opportunities for the parasite to escape vaccine‐induced immunity. In that respect, we have proposed TSA‐1 antigen as an additional component of our vaccine (de la Cruz et al., 2019; Dumonteil et al., 2004; Quijano‐Hernández et al., 2013; Villanueva‐Lizama et al., 2018), which was also found to be highly conserved among *T. cruzi* DTUs (Knight et al., 2014).

Nevertheless, this study has some limitations, the main one being that strain diversity may be further expanded as mentioned above, particularly for non‐TcI parasite strains, as additional sequence variants may be present in these DTUs as well as from some of the less represented countries from our study. Further genotyping of Tc24 antigens from strains currently circulating in Chagasic patients across the Americas should help expand our study.

In conclusion, we have demonstrated that Tc24 antigen is highly conserved in parasite strains originating from a wide geographic range in the Americas and covering DTUs TcI to TcVI. In addition, diversifying selection pressure was restricted to a few residues, which would limit immune evasion, and most of the protein was under strong purifying selection. This was likely associated, at least in part, with functional/structural constrains on the protein. These results indicate that Tc24 is an excellent vaccine candidate, which would be effective against a wide diversity of *T. cruzi* parasite strains across the continent. Further development of this vaccine candidate should represent a scientific and public health priority.

## Supporting information

Figure S1

Table S1

## Data Availability

Sequence data for this study are available at the TriTryp (https://tritrypdb.org/tritrypdb/) and SRA (https://www.ncbi.nlm.nih.gov/sra) databases.
