# Supplementary material for: Extent of polymorphism and selection pressure on the Trypanosoma cruzi vaccine candidate antigen Tc24
Source: Evol Appl. 2020 Sep 10;13(10):2663–72. doi: 10.1111/eva.13068 (PMC7691455; doi:10.1111/eva.13068)
Supplement: Supplementary file 1 — Figure S1 [file EVA-13-2663-s001.docx]

**SUPPLEMENTARY MATERIALS**


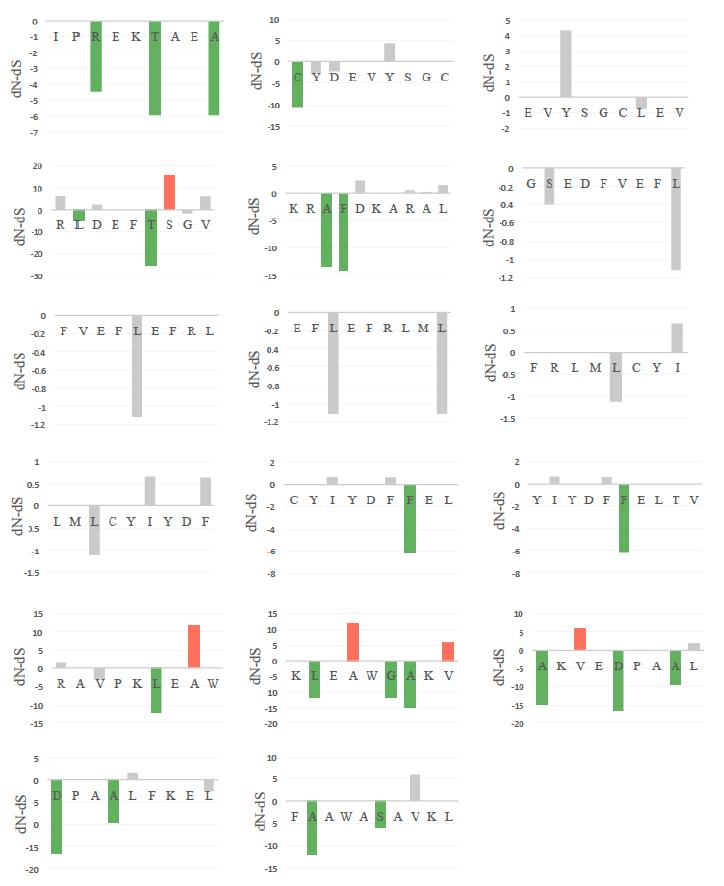


**Supplementary Figure S1:** Detail of selective pressures (dN-dS ratio) on the Tc24 protein predicted epitopes. Selective pressures on each epitope is expressed as dN-dS ratio as determined by SLAC analysis. Statistically significant selection pressure is highlighted in green (purifying selection) and red (diversifying selection), respectively.
