# Supplementary material for: Extent of polymorphism and selection pressure on the Trypanosoma cruzi vaccine candidate antigen Tc24
Source: Evol Appl. 2020 Sep 10;13(10):2663–72. doi: 10.1111/eva.13068 (PMC7691455; doi:10.1111/eva.13068)
Supplement: Supplementary file 2 — Table S1 [file EVA-13-2663-s002.docx]

**SUPLEMENTARY MATERIALS**

**Supplementary Table S1: Selection pressure on the Tc24 protein.**

| Codon | Nucleotide position | Polymorphism | dS | dN | *p* value |
| --- | --- | --- | --- | --- | --- |
| 18 | 54 | GAT->GAC | 6.707 | 0.000 | Neg. 0.001 |
| 33 | 99 | CGC->CCC | 0.000 | 9.500 | Pos. 0.000 |
| 40 | 120 | CGT->CGC | 3.000 | 0.000 | Neg. 0.037 |
| 43 | 129 | ACC->ACG | 4.000 | 0.000 | Neg. 0.012 |
| 46 | 138 | GCA->GCG | 4.000 | 0.000 | Neg. 0.012 |
| 47 | 141 | AAA->AAG | 9.126 | 0.000 | Neg. 0.001 |
| 49 | 147 | CGC->CGT | 5.000 | 0.000 | Neg. 0.004 |
| 51 | 153 | ATC->ATT | 5.659 | 0.000 | Neg. 0.003 |
| 66 | 198 | TGC->TCG/TGT/TTC | 8.258 | 1.146 | Neg. 0.012 |
| 80 | 240 | TTG->CTG | 3.316 | 0.000 | Neg. 0.031 |
| 84 | 252 | ACG->ACC | 17.000 | 0.000 | Neg. 0.000 |
| 85 | 255 | CCG->TCG | 0.000 | 10.342 | Pos. 0.000 |
| 87 | 261 | GTG->CTG | 0.000 | 4.018 | Pos. 0.038 |
| 94 | 282 | GCA->GCG | 9.000 | 0.000 | Neg. 0.000 |
| 95 | 285 | TTC->TTG/TTT | 10.206 | 0.797 | Neg. 0.002 |
| 130 | 390 | TTC>TTT | 4.139 | 0.000 | Neg. 0.026 |
| 136 | 408 | TTC->TTT | 3.446 | 0.000 | Neg. 0.037 |
| 139 | 417 | ATT->ATC | 4.714 | 0.000 | Neg. 0.006 |
| 152 | 456 | TTC->CTC/GTC | 0.000 | 6.866 | Pos. 0.038 |
| 159 | 477 | CTT->CTC | 8.000 | 0.000 | Neg. 0.000 |
| 161 | 483 | GCG->CAG | 0.000 | 7.838 | Pos. 0.003 |
| 163 | 489 | GGC->GGT | 8.000 | 0.000 | Neg. 0.000 |
| 164 | 492 | GCC->GCG/GCA | 10.000 | 0.000 | Neg. 0.000 |
| 166 | 498 | GTC->ATC | 0.000 | 3.897 | Pos. 0.048 |
| 168 | 504 | GAT->GAC | 11.065 | 0.000 | Neg. 0.000 |
| 171 | 513 | GCG->GAG/GCA | 7.360 | 0.925 | Neg. 0.007 |
| 177 | 531 | GAT->GAC | 2.924 | 0.000 | Neg. 0.052 |
| 179 | 537 | AAC->AAT | 10.228 | 0.000 | Neg. 0.000 |
| 182 | 546 | GGG->GGT | 4.000 | 0.000 | Neg. 0.012 |
| 186 | 558 | TTC->TTT | 7.214 | 0.000 | Neg. 0.001 |
| 190 | 570 | GCT->GCA | 8.000 | 0.000 | Neg. 0.000 |
| 194 | 582 | TCT->TCG | 4.000 | 0.000 | Neg. 0.013 |
| 196 | 588 | GTC->CGC | 0.000 | 4.000 | Pos. 0.039 |
| 205 | 615 | GAC->GAT | 6.162 | 0.000 | Neg. 0.004 |
| 210 | 630 | AGC->AGT | 15.296 | 0.000 | Neg. 0.000 |
